# Enhanced production of individual ganoderic acids by integrating *Vitreoscilla* haemoglobin expression and calcium ion induction in liquid static cultures of *Ganoderma lingzhi*

**DOI:** 10.1111/1751-7915.13381

**Published:** 2019-02-28

**Authors:** Jun‐Wei Xu, Tong‐Hui Yue, Xuya Yu, Peng Zhao, Tao Li, Na Li

**Affiliations:** ^1^ Faculty of Life Science and Technology Kunming University of Science and Technology Kunming 650500 China; ^2^ Faculty of Science Kunming University of Science and Technology Kunming 650500 China

## Abstract

Ganoderic acids produced by *Ganoderma* exhibit anticancer and antimetastatic activities. A novel approach by combining *Vitreoscilla* haemoglobin (VHb) expression and calcium ion induction was developed to enhance ganoderic acid (GA) production in liquid static cultures of *G. lingzhi*. The maximum contents of GA‐O, GA‐S and GA‐Me were 1451.33 ± 67.50, 1431.23 ± 79.74 and 1283.81 ± 85.13 μg per 100 mg cell weight, respectively under the integrated approach, which are the highest contents as ever reported in *Ganoderma*. The contents of squalene and lanosterol were increased by 2.0‐ and 3.0‐fold in this case compared with those in the control. The transcription levels of 3‐hydroxy‐3‐methylglutaryl coenzyme A reductase, farnesyl‐diphosphate synthase, squalene synthase and cytochrome P450 CYP5150L8 were upregulated by 2.56‐, 3.31‐, 2.59‐ and 6.12‐fold respectively. Additionally, the expression of VHb improved the ratio of type I to type II GA in liquid static cultivation of *G. lingzhi*. The transcription levels of *cyp512a2*,* cyp512v2* and *cyp512a13*, candidate cytochrome P450 genes involved in oxidative modification of the lanostane skeleton in GA biosynthesis, were also increased by 2.28‐, 2.65‐ and 3.54‐fold in the VHb‐expressing strain respectively. Our results illustrated that the approach described here efficiently improved GA production in *G. lingzhi* fermentation.

## Introduction

**Figure 1:**
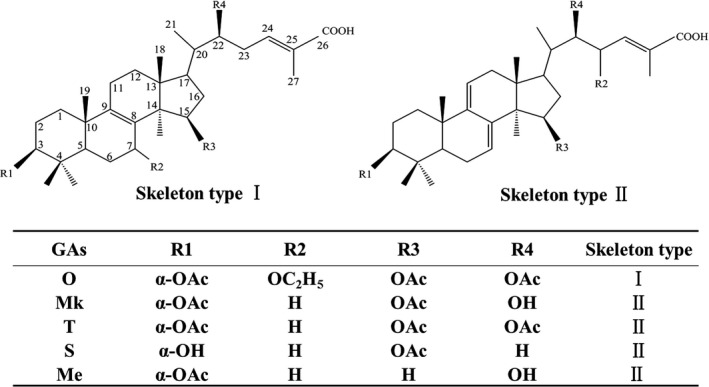
Chemical structures of GA‐O, GA‐Mk, GA‐T, GA‐S and GA‐Me.

*Ganoderma lingzhi* has been used to prevent and treat various human diseases for thousands of years in East Asia (Paterson, 2006). Ganoderic acids (GAs), highly oxygenated C30 lanostane‐type triterpenoids produced by *Ganoderma* species*,* have a variety of important pharmacological activities. For instance, Ganoderic acid (GA)‐Mk and GA‐S induce the apoptosis of HeLa cells (Liu and Zhong, 2011; Liu *et al*., 2012). GA‐T and GA‐Me inhibit lung cancer cell growth and metastasis (Tang *et al*., 2006; Wang *et al*., 2007), and GA‐O has cytotoxicity against lung and HeLa cancer cells (Wang *et al*., 2010). GAs have two types of carbon skeletons (Fig. 1): GA‐Mk, GA‐S, GA‐T and GA‐Me (type II) possess two conjugated double bonds on the tetracyclic rings, while GA‐O (type I) has only one double bond on the lanostane skeleton and more oxidative modification at C‐3, C‐7, C‐15 and C‐22 (Li *et al*., 2013).

Ganoderic acids are synthesized via the mevalonate/isoprenoid (MVA) pathway in *Ganoderma* species (Xu *et al*., 2010b). Lanosterol, an important intermediate, is converted to different individual GAs through a series of modifications, including oxidation, reduction and acylation reactions, in *Ganoderma lucidum* (Xu *et al*., 2010b; Chen *et al*., 2012; Zhang *et al*., 2017a,2017b). Some key genes involved in the upstream cyclization pathway of the lanostane skeleton, including 3‐hydroxy‐3‐methylglutaryl coenzyme A reductase (HMGR), farnesyl‐diphosphate synthase (FPS) and squalene synthase (SQS), have been cloned from *G. lucidum* (Shi *et al*., 2010; Zhou *et al*., 2014). Recently, the cytochrome P450 monooxygenase CYP5150L8 gene, which catalyses the three‐step oxidation of lanosterol at C‐26 in GA biosynthesis, was also been isolated and characterized (Wang *et al*., 2018). Other cytochrome P450 genes, such as *cyp512a2*,* cyp512v2* and *cyp512a13*, might be involved in the oxidative modification of the lanostane skeleton to form different individual GAs in *Ganoderma* (Chen *et al*., 2012; Jiang *et al*., 2018; Yang *et al*., 2018).

Due to their important pharmacological activities, many strategies have been used to increase the production of GAs during mycelia fermentation. Progress has been achieved in the last several decades in enhancing GA production in *G. lucidum* by the manipulation of fermentation strategies (Xu *et al*., 2010a; Upadhyay *et al*., 2014; Wang *et al*., 2016), addition of metal ions and elicitors (Ren *et al*., 2010; Xu and Zhong, 2012; You *et al*., 2013, 2017; Zhang *et al*., 2014; Gu *et al*., 2018) and genetic engineering (Xu *et al*., 2012; Xu and Zhong, 2015; Li *et al*., 2016a,2016b,2016c; Zhang *et al*., 2017a,2017b). However, further improvement of GA production is necessary to meet the demand of increasing preclinical studies and commercial applications.

The combination of both a good producer strain and an efficient bioprocess strategy is a promising approach for the enhancement of secondary metabolites. For example, the integration of precursor feeding and tryptophan decarboxylase gene overexpression leads to a higher accumulation of indole alkaloids than either strategy alone in *Catharanthus roseus* (Canel *et al*., 1998; Whitmer *et al*., 2002). In *Salvia miltiorrhiza*, it is more effective to combine elicitor treatments with transgenic technology for high enhancement of tanshinone content (Hao *et al*., 2015). However, the effect of integrating genetic modification and fermentation strategies in the production of secondary metabolites has not been studied in *Ganoderma*. A liquid static cultivation strategy is effective in promoting the production of GAs by *G. lucidum* compared with liquid shaking culture (Xu *et al*., 2010a). Xu *et al*. reported that calcium ion addition improved GA production in a liquid static culture of *G. lucidum* (Xu and Zhong, 2012). Our recent work found that the heterologous expression of the *Vitreoscilla* haemoglobin (VHb) gene increased GA biosynthesis in submerged cultivation of *G. lucidum* (Li *et al*., 2016a). Therefore, in this study, we observed the effects of VHb expression and calcium ion induction on GA biosynthesis in a liquid static culture of *G. lingzhi*.

Many secondary metabolites have similar chemical structures but are bioactively different (Zhong and Yue, 2005). Therefore, the distribution of secondary metabolites is of great interest for biotechnological applications. External environmental factors and genetic manipulation usually play important roles in regulating the heterogeneity of secondary metabolites. For instance, the ratio of the protopanaxatriol‐type to the protopanaxadiol‐type of ginsenosides can be altered by changing oxygen pressure and with the addition of phenobarbital in cell cultures of *Panax notoginseng* (Han and Zhong, 2003; Yue and Zhong, 2008). In *Aurantiochytrium sp*., heterologous expression of the VHb gene increases the ratio of astaxanthin to beta‐carotene significantly (Suen *et al*., 2014). In *G. lucidum*, Zhang *et al*. reported that enriching oxygen level affects the individual GA distribution in a liquid static culture (Zhang and Zhong, 2010). Liang *et al*. found that phenobarbital altered the individual GA distribution in a liquid static culture of *G. lucidum* (Liang *et al*., 2010). However, the effect of genetic modification of *Ganoderma* on individual GA distributions has not been investigated yet.

In this work, an integrated approach by combining VHb expression and calcium ion induction was applied to enhance the production of individual GAs in a liquid static culture of *G. lingzhi*. Moreover, the effect of heterologous expression of the VHb gene on individual GA distributions was also analysed in *G. lingzhi*.

## Results and discussion

### VHb expression enhanced the production of individual GAs in liquid static cultures of *G. lingzhi*

**Figure 2:**
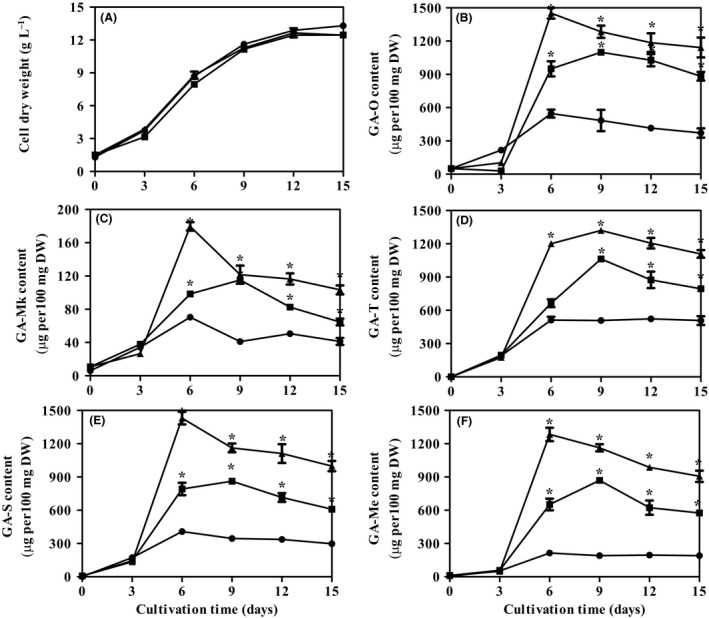
Time profiles of the cell growth (A) and contents of GA‐O (B), GA‐Mk (C), GA‐T (D), GA‐S (E) and GA‐Me (F) in liquid static culture of wild‐type *G. lingzhi* (filled circle), VHb‐expressing strain (filled square) and VHb‐expressing strain with calcium ion induction (filled triangle) respectively. *Significantly difference from value for the wild‐type *G. lingzhi* in the liquid static culture mode (*P *<* *0.05).

The kinetics of cell growth of the wild‐type (WT) and VHb‐expressing *G. lingzhi* (VHb) in liquid static culture conditions are shown in Fig 2A. No significant difference was observed between WT and VHb in the liquid static culture mode. This may be due to the sufficient supply of oxygen and a relatively low cell density in the WT and VHb‐expressing strain under the normal culture condition (Li *et al*., 2016a).

GA‐O, GA‐T, GA‐Me, GA‐S and GA‐Mk were detected as major GA components from *G. lingzhi* mycelia (Xu *et al*., 2010a; Wang *et al*., 2012). The time profiles of GA‐O, GA‐Mk, GA‐T, GA‐S and GA‐Me accumulation in both WT and VHb cultures are shown in Fig. 2B–F. In the WT, the contents of GA‐T and GA‐Me increased until day 6, then their contents remained approximately constant; GA‐Mk, GA‐S and GA‐O reached their maximum values on day 6 and slightly decline thereafter. For the VHb strain, the contents of these five major individual GAs increased significantly and reached their maximal levels at day 9, followed by a decrease until the end of fermentation. The maximum concentrations of GA‐O, GA‐Mk, GA‐T, GA‐S and GA‐Me obtained in VHb cultures were 1098.92 ± 7.74, 115.22 ± 3.24, 1062.83 ± 26.89, 861.28 ± 4.70 and 869.61 ± 20.42 μg per 100 mg cell dry weight (CDW), respectively, which were about 2.01, 1.64, 2.03, 2.11 and 4.05 times those in the WT. Our results showed that the expression of VHb effectively improved the production of individual GAs in liquid static cultures of *G. lingzhi*.

### Further improvement in production of individual GAs in liquid static cultures of VHb‐expressing *G. lingzhi* by calcium ion induction

The time profiles of the contents of GA‐O, GA‐Mk, GA‐T, GA‐S and GA‐Me of the VHb strain with 10 mM calcium ion induction condition are shown in Fig. 2B–F. The contents of GA‐O, GA‐Mk, GA‐S and GA‐Me increased rapidly from day 3 to day 6 and then decreased slightly until the end of fermentation. The GA‐T content increased significantly and reached a maximal value at day 9; afterwards, there was a slight decrease in the liquid static culture. The maximum contents of GA‐O, GA‐Mk, GA‐T, GA‐S and GA‐Me obtained here were 1451.33 ± 67.50, 179.66 ± 5.06, 1320.59 ± 20.84, 1431.23 ± 79.74 and 1283.81 ± 85.13 μg per 100 mg CDW, respectively, which were increases of 32%, 56%, 24%, 66% and 48% compared with VHb alone. In particular, the contents of GA‐O, GA‐S and GA‐Me obtained under the integrated approach were much higher than previously reported highest levels, including those using static liquid of the WT (Wang *et al*., 2010), phenobarbital induction (Liang *et al*., 2010) and higher oxygen levels (Zhang and Zhong, 2010). These results suggest that combining genetic engineering and the fermentation strategy can more efficiently improve the production of individual GAs in *G. lingzhi*. Similar results have also been reported for *Streptomyces graminearus*, in which combined gene cluster engineering and glycine feeding increase gougerotin production by 2.5‐, 1.25‐,and 1.3‐fold higher compared to the WT strain, the engineered strain and the WT strain by glycine feeding respectively (Jiang *et al*., 2013). Exogenous feeding of nutrients to cultures of the engineered *S. tsukubaensis* leads to 3.2‐, 1.3‐ and 2.6‐fold improvement in FK506 production compared with those in the WT strain, the engineered strain and the WT strain fed with nutrients respectively (Huang *et al*., 2013).

### Effects of the integrated approach on the accumulation of intermediates and on GA biosynthetic gene expression

**Figure 3:**
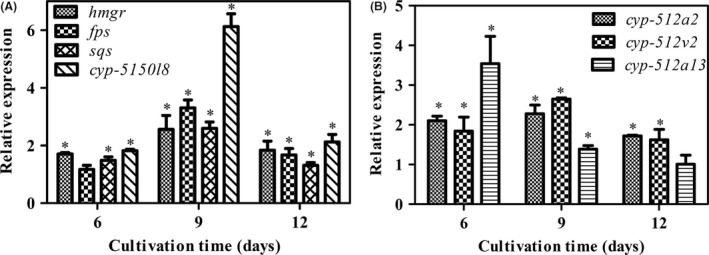
Transcription levels of *hmgr*,* fps*,* sqs* and *cyp5150 l8* in liquid static cultures of wild‐type *G. lingzhi* and VHb‐expressing strain with calcium ion induction (A). Transcription levels of *cyp512a2*,* cyp512v2* and *cyp512a13* in liquid static cultures of wild‐type *G. lingzhi* and VHb‐expressing strain (B). A expression level of the samples from the wild‐type strain is defined as 1.0, and the expression levels under other cultivation strategies are expressed as fold changes over the reference sample. Symbols are the same as those in Fig. 2.

Table 1 shows that the maximum squalene content with the integrated approach was 1.50 ± 0.28 μg g CDW^−1^ on day 9, which was 2.0‐fold higher than that under control conditions. Although the content gradually decreased to 0.50 ± 0.28 μg g CDW^−1^ on day 12, it still maintained a higher value than the control. The concentration of lanosterol with the integrated approach reached a maximum of 106.55 ± 2.74 μg g CDW^−1^ on day 9, but declined to 81.74 ± 1.80 μg g CDW^−1^ on day 12. The maximum level of lanosterol with the integrated approach was 1.4 times higher than that under the control conditions. Fig. 3A shows that the maximum transcription levels of *hmg*,* fps*,* sqs* and *cyp‐5150 l8* observed on day 9 under the integrated approach were 2.56, 2.59, 3.31 and 6.12 times higher than those in the control conditions respectively. These values decreased from day 9 to day 12, but still maintained higher levels than those in the control.

**Table 1:** Contents of squalene and lanosterol in liquid static cultures of wild‐type *G. lingzhi* (the control) and VHb‐expressing strain with calcium ion induction (the integrated approach)

Culture conditions	Cultivation time	Biomass (g l^−1^)	Squalene content (μg g DCW^−1^)	Lanosterol content (μg g DCW^−1^)
The control	Day 6	8.70 ± 0.48	0.30 ± 0.06	34.69 ± 7.59
The integrated approach		8.78 ± 0.57	0.67 ± 0.22*	88.21 ± 2.51*
The control	Day 9	11.62 ± 0.39	0.75 ± 0.12	75.18 ± 1.22
The integrated approach		11.28 ± 0.16	1.50 ± 0.28*	106.55 ± 2.74*
The control	Day 12	12.88 ± 0.16	0.34 ± 0.02	67.67 ± 5.26
The integrated approach		12.65 ± 0.72	0.50 ± 0.03*	81.74 ± 1.80*

* Significantly different from value for the control (*P* < 0.05).

The contents of lanosterol and squalene and the expression of GA biosynthetic genes were higher under the integrated approach compared to the control liquid static culture of *G. lingzhi*. These trends were positively correlated with the enhanced production of individual GAs, which indicated that that the improved GA production could be attributed to increased precursor supply and the upregulated expression of biosynthetic genes. In *Antrodia cinnamonea* and *Paraconiothyrium,* a higher supply of precursors increases the production of triterpenoids and diterpene respectively (Chen *et al*., 2016; Solima *et al*., 2017). Previous reports have also found that there is a positive correlation between the expression levels of triterpene biosynthetic genes and the contents of ginsenoside and astragalosides (Jiao *et al*., 2016; Li *et al*., 2016b). This was also observed in our previous works with squalene synthase overexpression and the integration of nitrogen limitation and calcium ion addition (Zhou *et al*., 2014; Li *et al*., 2016c).

### VHb expression increased the ratio of type I to type II GAs in static liquid cultures of *G. lingzhi*

As shown in Table 2, the ratios of type I to type II GAs were 0.422 and 0.432 in the VHb strain, while the ratios were 0.375 and 0.358 in the WT of *G. lingzhi* on day 12 and day 15 respectively. VHb expression thus increased the ratio of type I to type II GAs in static liquid cultures of *G. lingzhi*. Fig. 3B shows that the transcription levels of *cyp‐512a2* and *cyp‐512v2* in the VHb strain were 2.28‐ and 2.65‐fold higher than those in the WT on day 9. For *cyp‐512a13*, the transcription level in the VHb strain was 3.54‐fold higher than that in the WT on day 6. The transcription levels of these P450 genes were thus significantly induced by VHb compared to the WT.

**Table 2:** The effect of heterologous expression of the VHb on individual GA distributions in liquid static cultures of *G. lingzhi*. WT, wild‐type *G. lingzhi*; VHb, VHb‐expressing *G. lingzhi*

Cultivation time	Strains	GA content (μg/100 mg CDW)		Type I: Type II GAs
		Type I GAs	Type II GAs	
Day 12	WT	415.08 ± 24.28	1106.32 ± 21.53	0.375
	VHb	1028.51 ± 95.60	2434.54 ± 106.22	0.422
Day 15	WT	371.33 ± 73.40	1037.17 ± 124.73	0.358
	VHb	883.36 ± 54.71	2044.27 ± 131.89	0.432

Oxidative modification of the lanostane skeleton plays an important role in the biosynthesis of GA. The increased ratio of type I to type II GAs may be due to the stimulated activities of oxygenase involved in the GA biosynthesis pathway (Stark *et al*., 2015; Li *et al*., 2016a). The transcription levels of *cyp512a2*,* cyp512v2* and *cyp512a13* were upregulated in the VHb strain, suggesting that the increased ratio may be related to higher transcription levels of those cytochrome P450 genes. In *P. notoginseng*, the induced activity of the cytochrome P450 enzyme protopanaxadiol 6‐hydroxylase increases the ratio of protopanaxatriol‐type to protopanaxadiol‐type ginsenoside (Yue and Zhong, 2008). The detailed roles of these cytochrome P450s in regulating GA distribution need to be further investigated in the future.

In summary, we demonstrated that an integrated approach that combines VHb expression and calcium ion induction can effectively improve the production of individual GAs in liquid static cultivation of *G. lingzhi*. In addition, the expression of the VHb gene increased the ratio of type I to type II GAs in liquid static cultures of *G. lingzhi*. This work will be useful for large‐scale production of individual GAs and to understand the regulatory mechanisms of GA heterogeneity in *G. lingzhi*.

## Experimental procedures

### Strains and culture conditions

*Ganoderma lucidum* CGMCC 5.616 was bought from the China General Microbiological Fermentation Center and is conspecific with *Ganoderma lingzhi* (Zhang *et al*., 2017a,2017b). The VHb‐expressing *G. lingzhi* was constructed in a previous study (Li *et al*., 2016a). The pre‐culture and fermentation details of the liquid static culture of *G. lingzhi* were described before (Xu *et al*., 2010a) and are shown in the Appendix S1. CaCl_2_ was dissolved in water and was sterilized by filtering through 0.22‐μm polyvinylidene difluoride syringe filters (Millipore, Germany) before their additions into cultures. 10 mM of CaCl_2_ was added when shaking flask cultures were converted to liquid static cultures in 10‐cm‐diameter plate for incubation.

### Sampling, and measurements of cell dry weight, individual GAs, squalene and lanosterol

For sampling, three plates were taken each time. Cell dry weight was measured by a gravimetric method. Individual GAs, squalene and lanosterol were extracted and determined by methods described earlier (Xu *et al*., 2012; Zhou *et al*., 2014) (see also Appendix S1).

### Total RNA extraction and cDNA synthesis

The total RNA from *G. lingzhi* was extracted with TRIzol Reagent (Invitrogen, Carlsbad, CA, USA) and treated with DNase I to remove residual DNA. One microgram of total RNA was reverse‐transcribed by using the PrimeScript^TM^ RT reagent Kit (TaKaRa, Dalian, China).

### Isolation of cytochrome P450 genes from *G. lingzhi*

Partial cDNAs of cytochrome P450 monooxygenases CYP‐5150L8, CYP512 A2, CYP512 V2 and CYP512 A13 were isolated by PCR amplification using primers shown in Table S1. The amplified fragments were ligated into the pMD 19‐T Simple Vector by the T4 DNA ligase system (TaKaRa) and sequenced using both M13 +  and M13− as primers (Sangon, Shanghai, China).

### Real‐time quantitative PCR (qRT‐PCR) analysis

The transcription levels of *hmgr*,* fps*,* sqs*,* cyp‐5150 l8*,* cyp‐512a2*,* cyp‐512v2* and *cyp‐512a13* were measured by qRT‐PCR. The qRT‐PCR primer sequences of the 18S rRNA gene, *hmgr*,* fps* and *sqs* are described elsewhere (Xu *et al*., 2010a; Zhao *et al*., 2011). The qRT‐PCR primers for *cyp‐5150 l8*,* cyp‐512a2*,* cyp‐512v2* and *cyp‐512a13* are listed in Table S1. Real‐time PCR was performed using SYBR^®^ Premix Ex Taq™ II (TaKaRa) according to the manufacturer's instructions. The cDNA was quantified using a CFX96™ real‐time PCR detection system (Bio‐Rad, Inc., Hercules, CA, USA). The transcription levels of the target genes were normalized with that of the *G. lingzhi* 18S rRNA gene. A transcription level of 1 was defined for samples from the liquid static culture of the wild‐type strain, and the transcription levels in other cultivation strategies were expressed as fold changes of the mRNA levels over the reference level. Relative gene transcription levels were analysed according to the $2^{-\Delta \Delta C_{T}}$ method.

### Statistical analysis

All of the data were generated from three independent samples. Data are expressed as average values ± standard deviation. Statistical analysis was performed using Student's paired *t*‐test. The difference was considered significant when *P *<* *0.05 in a two‐tailed analysis.

## Conflicts of interest

None declared.

## Supporting information


**Appendix S1**. Culture of *Ganoderma lingzhi*, and analysis of individual ganoderic acids, squalene and lanosterol.
**Table S1**. Sequences of primers for PCR amplification and qRT‐PCR assay.
