# Supplementary material for: Enhanced production of individual ganoderic acids by integrating Vitreoscilla haemoglobin expression and calcium ion induction in liquid static cultures of Ganoderma lingzhi
Source: Microb Biotechnol. 2019 Feb 28;12(6):1180–7. doi: 10.1111/1751-7915.13381 (PMC6801144; doi:10.1111/1751-7915.13381)
Supplement: Supplementary file 1 — Appendix S1. Culture of Ganoderma lingzhi, and analysis of individual ganoderic acids, squalene and lanosterol. Table S1. Sequences of primers for PCR amplification and qRT‐PCR assay. [file MBT2-12-1180-s001.docx]

**Support information**

**1. Culture of *Ganoderma lingzhi***

The strain of *G. lingzhi* was maintained on potato-agar-dextrose slants. The slant was inoculated and incubated at 28 °C for 7 days. Preculture medium consisted of the

following components (g/L): glucose, 35; peptone, 5; yeast extract, 2.5; KH_2_PO_4_·H_2_O, 1; MgSO_4_·7H_2_O, 0.5; and vitamin B_1_, 0.05. Fermentation medium consisted of the following components (g/L): lactose, 35; peptone, 5; yeast extract, 5; KH_2_PO_4_·H_2_O, 1; MgSO_4_·7H_2_O, 0.5; and vitamin B_1_, 0.05. For the first preculture, 40 ml of medium at an initial pH of 5.5 was prepared in a 250-ml flask, and then 10 ml of mycelium suspension from a slant culture was inoculated, followed by incubation for 5 days at 30 ^o^C on a rotary shaker (120 rpm). For the second preculture, 45 ml of medium was prepared in a 250-ml flask and inoculated with 5 ml of preculture broth (with ca. 600 to 700 mg dry weight/L), followed by 3 days of incubation at 30 ^o^C on a rotary shaker (120 rpm). For the fermentation, a 45 ml medium in a 250-ml flask was inoculated with 5 ml of second-stage preculture broth (with ca. 330 mg dry weight/L). The culture was incubated in the dark at 30 ^o^C on a rotary shaker at 120 rpm. After 3 days of shake-flask fermentation, the flasks were incubated statically at 30 ^o^C. Each plate contained 50 mL broth.

**2. Extraction and analysis of individual GAs**

For individual GAs, the dried mycelia (100 mg) were extracted by 75 % (v/v) ethanol (3 mL), ultrasonic treatment for 1 h (three times). The supernatants were dried at 50 ^o^C under vacuum and redissolved in 500 μl absolute ethanol for high performance liquid chromatography (HPLC) analysis. The elution was performed at a flow rate of 1.0 mL/min with a linear gradient of solvent A (methanol/acetic acid, 100:0.5, v/v) and solvent B (water). The gradient, starting at sample injection, was linear from 80 % to 100 % A in 20 min. Chromatographic peaks were identified by comparing the retention times and spectra against the known standards. The standards of GA-T, GA-Me, GA-S and GA-O and GA-Mk were extracted and purified from mycelia with preparative liquid chromatography with purity over 99 %.

**3. Extraction and analysis of squalene and lanosterol**

Dried mycelia (50 mg) were saponified by 2 ml of 10 % (wt/vol) KOH–75% (v/v) ethanol solution at 50 ^o^C for 2 h. The mixture was extracted with 2 ml of hexane for three times. The hexane layer was collected and evaporated to dryness under N_2_. The residue was dissolved in 0.5 ml of acetonitrile for subsequent high-pressure liquid chromatography (HPLC) analysis. Chromatographic peaks were identified by comparing the retention times and spectra against the standards of squalene (≥98%, Sigma) and lanosterol (≥97%, Sigma, St. Louis, MO).

**Table S1 Sequences of primers for PCR amplification and qRT-PCR assay**

| Target gene | Primer sequences for  PCR amplification(5’-3’) | Primer sequences for  qRT-PCR assay (5’-3’) |
| --- | --- | --- |
| *cyp-5150l8* | CATCGTTATCTCGTGCGG | GGACAACCTCCCAAGCC |
|  | TAGGTTTTGGGATTGGGC | GCCCGATGAACAGAGTGAA |
| *cyp-512a2* | GAAGCCCAACGAAGACG | ATGACATCCTCAACTCCCTCG |
|  | CGGACCATCAGCCCCA | GACCATCAGCCCCAATACTCT |
| *cyp-512v2* | TGGAACATCAGCCCCTTGG | GGGAATGAATGGGTCGCTAT |
|  | CGGTCCTTCGTCTGCTTTAC | CGGTCCTTCGTCTGCTTTACTG |
| *cyp-512a13* | GTCTATCGTTCGTGGCTG | TCATCAGGGAGAAGCTAACACG |
|  | GGTTGTAGGAAGGATGGTA | CGGAAGCCCCACAAAGAC |
